# Supplementary material for: “So sometimes, it looks like it’s a neglected ward”: Health worker perspectives on implementing kangaroo mother care in southern Malawi
Source: PLoS One. 2020 Dec 17;15(12):e0243770. doi: 10.1371/journal.pone.0243770 (PMC7746165; doi:10.1371/journal.pone.0243770)
Supplement: S2 File — (DOCX) [file pone.0243770.s002.docx]

# Healthcare workers experiences with interventions to improve neonatal health

## Topic Guide

- *Introduce yourself and ask the participant how they are doing today, etc.*
- *Introduce the project and go through the consent form with the participant*
- *Get the participant to fill out the demographics form*

| Topic | Question | If not already brought up by participant, probe: |
| --- | --- | --- |
| Introduction | 1. How long have you worked in the unit? |  |
|  | 1. Which units do you prefer to work in? |  |
|  | 1. Please describe a typical day in this ward for you | - What do you do? - What are your responsibilities? |
| Training | 1. First of all, can you tell me more about when you started using KMC? |  |
|  | 1. Can you tell me how you were trained? | - Formal or informal? - Length of training? - Who were the trainers? |
|  | 1. In your training, what did you find most helpful/useful for using KMC in your workplace? |  |
|  | 1. What other things would you like to learn to better be able to use KMC in your workplace? |  |
|  | 1. What stood out for you while learning about KMC such as anything you found surprising or interesting? |  |
| Initiation | 1. I have a couple general questions about using KMC for newborn babies at your workplace. Would you describe to me how KMC is started at your workplace? | - Who has the authority to start? - Who actually starts it? - Any differences between shifts (day/night/weekend/holidays)? |
|  | 1. Would you describe factors that make it easier to start KMC? Can you provide an example? |  |
|  | 1. Would you describe factors that make it more difficult to start KMC? Can you provide an example? |  |
|  | 1. What do you do when you face challenges? Can you provide an example? | - Sources of support? - Shift differences? |
|  | 1. Would you describe any disagreements with initiating KMC that you may have observed or heard about? | - Who disagreed with who? - Why did they disagree? - What happened? - How was it resolved? - Any other examples? |
|  | 1. Can you describe a time at your workplace when there was a delay in starting KMC? | - How long was the delay? - Why do you think this happened? - What resulted in your example? |
|  | 1. Can you describe a time at your workplace when KMC was not given? | - Why? - What happened? |
| Monitoring | 1. Would you describe how monitoring happens at your workplace? | - Who monitors? - When and how often? - What do they do to monitor? - Where do they monitor? - Ideal vs reality? - Shift differences? |
|  | 1. Would you describe factors that make it easier to start KMC? Can you provide an example? |  |
|  | 1. Would you describe factors that make it more difficult to start KMC? Can you provide an example? |  |
|  | 1. What do you do when you face challenges? Can you provide an example? | - Sources of support? - Shift differences? |
|  | 1. Would you describe any disagreements with monitoring KMC that you may have observed or heard about? | - Who disagreed with who? - Why disagree? - What happened? - How was it resolved? - Any other examples? |
|  | 1. Can you describe a time at your workplace when there was a delay in monitoring? | - How long? - Why? - What happened? |
|  | 1. Can you describe a time at your workplace when monitoring did not happen? | - Why? - What happened? |
| Perceptions of health care professionals | 1. Would you describe how low birthweight babies were managed in his workplace before KMC? |  |
|  | 1. What has changed since KMC was introduced? |  |
|  | 1. In your experience, can you describe situations where KMC helped the baby get well? | - What happened? - Why they think it happened? - What could be learned from this situation? |
|  | 1. In your experience, can you describe situations where KMC caused harm to the baby? | - What happened? - Why they think it happened? - What could be learned from this situation? - Did it change your opinion on KMC? |
|  | 1. Overall, what do you think about KMC? | - How does using KMC make you feel? - What did you like about using it and what did you not like? - How has it influenced your practice? |
| Perceptions of parents | 1. What are some of the perceptions that mothers/guardians have of KMC? |  |
|  | 1. Why do you think they think this way? |  |
|  | 1. How do you explain KMC to the mother/guardian? | - Who talks to guardians? - When/at what point - Give an example of what you would say - If no one talks to the parents then why not? |
|  | 1. Any examples of parents who refused? Who, why and what was done? |  |
|  | 1. Any examples of parents who initially refused and then accepted? What changed their mind? |  |
|  | 1. Any examples of parents who accepted right away? Why do you think this was? |  |
|  | 1. Would you describe how parents care for their baby while on KMC? | - Holding, changing, feeding, - Challenges - How to support, peer support |
|  | 1. What factors do you think make it easier for parents to be comfortable with KMC? Can you provide an example? |  |
|  | 1. What factors do you think makes it more difficult for parents to use KMC? Can you provide an example? |  |
| Closing | 1. Thank you. These are all the questions I had for you. Is there anything you would like us to know about your experience with KMC or how health care workers could be supported to continue to provide KMC to newborns? |  |
