# Supplementary material for: “So sometimes, it looks like it’s a neglected ward”: Health worker perspectives on implementing kangaroo mother care in southern Malawi
Source: PLoS One. 2020 Dec 17;15(12):e0243770. doi: 10.1371/journal.pone.0243770 (PMC7746165; doi:10.1371/journal.pone.0243770)
Supplement: S3 File — (DOCX) [file pone.0243770.s003.docx]

## Ndondomeko yofotokoza maganizo aogwira ntchito mu chipatala paukadaulo wothandiza kutukula miyoyo yamakanda

- *Zifotokozeni nokha ndipo afunseni otenga nawo gawo mu kafukufukuyi kuti ali bwanji ndi zina zotero.*
- *Fotokozani za project ndipo awerengereni anthu zoyenera kudziwa mu kafukufukuyi ndipo pemphani chilolezo chawo potengapo nawo gawo mu kafuku fukuyi.*
- *Pemphani wotenga mbali kuti alembe ma fomu*

| **Mutu** | **Funso** | **Funsani izi ngati wotenga mbali sanalankhulepo:** |
| --- | --- | --- |
| **Chiyambi** | 1. Kodi mwagwira ntchito nthawi yayitali bwanji mu chipinda ichi? |  |
|  | 1. Kodi ndi zipinda ziti zomwe mumakhutitsidwa nazo kugwiramo ntchito? |  |
|  | 1. Kodi mungafotokoze za tsiku lanu mmene limayendera mchipindachi? | - Kodi mumagwira ntchito yanji? - kodi ma udindo anji omwe mumayenera ku kwanilitsa? |
| **Maphunziro** | 1. Choyamba tafotokozani zambiri za nthawi imene munayambira kugwiritsa ntchito ndondomekoyi ya KMC?(*Tchulani ndondomeko yofunikira)* |  |
|  | 1. Mungandifotokozere zamomwe munaphunzitsidwira za ndondomekoyi/thandizoli? | - Anali maphunziro a mkalasi kapena ayi? - Munaphunzira kwa nthawi   yayitali bwanji?   - Anakuphunzitsani anali yani? |
|  | 1. Mu maphunziro anu, Kodi ndichiti chomwe munachiwona chothandiza kapena chofunikira pogwiritsa ntchito ndondomekoyi/thandizoli la KMC pa ntchito yanu? | - Mukuganiza kuti ndi chiti chimene chinali chofunikira ndipo chosafunikira ku kuyikidwa mu maphunziro anu? |
|  | 1. Kodi ndi zinthu zina ziti zomwe munakakonda kuphunzira kuti zikuthandizireni pogwiritsa ntchito ndondomekoyi/thandizoli ya KMC pa ntchito yanu? |  |
|  | 1. Kodi ndi zinthu ziti zomwe zinali zopambana mmene mumaphunzira za ndondomeko kapena thandizoli la KMC? Monga zomwe munawona kuti zinali zosangalatsa kapena zodabwitsa. |  |
| **Kukhazikitsa** | 1. Ndili ndi mafunso angapo wokhuzana ndi ndondomekoyi kapena thandizo la KMC kwa ana ongobadwa kumene ku ntchito kwanu.   Kodi mungandifotokozere kuti kupereka thandizo la KMC zimayenda bwanji ku ntchito kwanu? | - Kodi ali ndi mphamvu yoyambitsa ndi ndani? - Kweni kweni amayambitsa ndi ndani? - Thandizo limaperekedwa liti kuchokera pa nthawi imene kwaperekedwa chiganizo? - Pali kusiyana kuli konse malingana ndi mthawi yantchtito monga : Tsiku/Usiku/ Kumapeto kwa sabata (week end)/ tchuti)? |
|  | 1. Mungafotokoze zifukwa zimene zimaphweketsa kapena kuthandizira kayambidwe ka ndondomeko ya KMC? Mungapereke chitsanzo? |  |
|  | 1. Mungafotokoze zifukwa zomwe zimavutitsa/limbitsa kayambidwe ka ndondomekoyi/ thandizoli la KMC? Mungapereke chitsanzo? |  |
|  | 1. Kodi mumatani mukakumana ndi ziphinjo? Mungapereke chitsanzo | - Thandizo mumalipeza kuti? - Kusiyana kulikonse malingana ndi nthawi ya ntchito?? |
|  | 1. Mungafotokoze kusamvetsetsana poyambitsa ndondomeko kapena thandizoli la KMC komwe mwawonapo kapena kumvapo? | - Amene samamvetsetsana anali ndani ndi ndani? - Sanamvetsetsani chifukwa chani? - Chinachitika nchani? - Anapanga bwanji chitsankho potsiriza? - Mungapereke Chitsanzo china? |
|  | 1. Mungafotokoze nthawi yimene panali kuchedwa kuyamba ndondomekoyi kapena thandizo la KMC ku ntchito kwanu? | - Munachedwa kwa nthawi yayitali bwanji? - Mukuganiza kuti Chinachitika ndichani kuti muchedwe? - Kodi zotsatila zake zinali zotani muchitsanzo chanuchi? |
|  | 1. Mungafotokoze nthawi yomwe kuntchito kwanu simunapereke ndondomekoyi kapena thandizoli la KMC? | - Chifukwa chiyani? - Chinachitika ndi chiyani? |
| **Kulondoloza** | 1. Mungafotokoze mmene kalondolondo wantchito yi amayendera ku ntchito kwanu? | - Amalondoloza ndi ndani? - Amapanga nthawi zanji ndipo mowilikiza bwanji? - Amalondoloza chani ndipo chifukwa chani? - Amalondoloza kuti? - Zoyenera kuchitika ndi Zochitika? - Pa kusinthana nthawi zogwirira ntchito? |
|  | 1. Mungafotokoze zifukwa zomwe zimathandiza kalondolondo wa ndondomekoyi kapena thandizolo la KMC? Mungapereke chitsanzo? |  |
|  | 1. Mungafototkoze Zifukwa zomwe zimavutitsa kapena kulimbitsa kalondolondo wa ndondomekoyi kapena thandizoli la KMC? Mungapereke Chitsanzo? |  |
|  | 1. Kodi mumatani mukakumana ndi ziphinjo? Mungapereke chitsanzo? | - Kodi thandizo lanu mumalipeza kuti? - Pa kusithana kwa nthawi zogwirira ntchito? |
|  | 1. Mungafotokoze kusamvetsetsana komwe polondoloza ndondomekoyi kapena thandizo la KMC komwe mwawonapo kapena kumvapo? | - Amene sanamvetsetsani anali ndani ndi ndani? - Sanamvetsetsane chifukwa chani? - Chinachitika nchani? - Zinatha bwanji? - Mungapereke zitsanzo china? |
|  | 1. Mungafotokoze nthawi yimene munachedwapo popanga kalondolondo wa ndondomekoyi kapena thandizoli la KMC? | - Munachedwa kwa nthawi yayitali bwanji? - Chifukwa chiyani?   Chinachitika ndi chiyani? |
|  | 1. Mungafotokoze nthawi yimene kalondolondo kapena thandizo sanachitike ku ntchito kwanu? | - Chifukwa chani? - Chinachitika ndichani? - Ndimowilikiza bwanji mmene wodwala salondolozedwa? |
| **Maonedwe/ma mvetsedwe a ogwira ntchito m’chipatala** | 1. Mungafotokoze mmene mwana wowonda kwambiri munakathanilana nalo ku ntchito kwanu ndondomekoyi ya KMC yisanabwere? |  |
|  | 1. Mukuonapo kusintha kwanji chiyambireni ndondomeko kaena chithandizo cha KMC? | - Mu zipatala za m’ma boma, ndondomekoyi kapena thandizoli lakhuzapo bwanji katumizidwe ka anthu ku chipatala cha Queens (referral)? |
|  | 1. Mu ukadawulo/luso lanu, mungafotokoze nyengo zomwe ndondomekoyi kapena thandizoli la KMC linathandiza mwana wakhanda kupeza bwino? | - Chinachitika nchani? - Akuganiza kuti china chitika nchani? - Kodi angatengedwepo phunziro lanji pamenepo? |
|  | 1. Muukadawulo wanu mungafotokoze nthawi zimene ndondomeko ya KMC chinapereka chiwophyezo kwa mwana wakhanda? | - Chinachitika nchani? - Mukuganiza kuti zinachitikika chifukwa chani? - Kodi ndi phunziro lanji lingatengedwe pazimenezi? - Kodi zinasintha maganizo amene munali nawo pa ndondomekoyi? |
|  | 1. Mophereza kodi mukuganiza bwanji za ndondomekoyi kapena thandizo la KMC? | - Kodi ndondomekoyi mumakumvetsani bwanji? - Kodi munakondapo chani mu mundondomekoyi? Ndipo ndi chiti chomwe simunachikonde mu ndondomekoyi? - Kodi zakuthandizani bwanji mu ntchito yanu? |
| **Mamvedwe/ Maonedwe a makolo** | 1. Kodi makolo kapena oyang’anira amayimvetsa bwanji ndondomeko kapena thandizo la KMC? | - What are cultural beliefs around components of the intervention (i.e. depending on intervention: breastmilk, colostrum, always holding newborn, blue lights, warming boxes, etc.)?   Kodi ndi zikhulupiliro zanji zomwe amakhala nazo zokhuzana ndi ndondomekoyi kapena thandizoli? Fufuzani maganizo awo pa zinthu izi: Mkaka wa mmawere, Mkaka woyamba kutuluka mmaere, mwana akabadwa, nthawi zones kufungatila ana ongobadwa kumene, magetsi ochotsa chikasu, chifunditso, ndi zina zotero)? |
|  | 1. Mukuganiza kuti nchifukwa chani amaganiza munjira yotere? | - Pali kuganizira kuli konse kutengera nkhani ya ka chirombo ka HIV kwa omwe ali wokhuzidwa? |
|  | 1. Kodi mu mawafotokozera bwanji makolo kapena osamalira za ndondomeko kapena thandizoli la KMC? | - Amayankhula ndi kholo ndi ndani? - Amawalankhula nthawi yanji ndipo chifukwa chani? - Perekani chitsanzo chamomwe mungayankhulire - Kodi kholo lo lingalandire bwanji wuthenga mukuperekawo? - Ngati palibe amene amayankhulana ndi khololo, nchifukwa chani zili choncho? |
|  | 1. Pali chitsanzo chakholo lomwe linakanapo? Ndi ndani, anakana chifukwa chani? Ndipo atakana pa nachitikako nchani? |  |
|  | 1. Pali chitsanzo cha kholo lomwe linakana poyamba kenako nkuvomera? China sinthitsa maganizo awo nchani? |  |
|  | 1. Chilipo chitsanzo cha kholo lomwe linavomera pompo pompo? Mukuganiza kuti chinachititsa zimenezi nchani? |  |
|  | 1. Mungafotokoze momwe makolo amasamalira ana awo akamalandira ndondomeko kapena thandizo la KMC? | - Kuwafungatira, Kuwasintha, kudyetsa. - Ziphinjo - Kuthandiza, amathandizana bwanji ndi anzawo? |
|  | 1. Ndizifukwa ziti zimene mukuganiza zimathandiza makolo kukhazikika kapena kumvetsa zachothandizo cha KMC ndi chani? Mungapereke chitsanzo? |  |
|  | 1. Ndizifukwa ziti zomwe mukuganiza kuti zimawavuta makolo pogwiritsa ntchito ndondomekoyi/ thandizoli? Mungapereke chitsanzo? | - Kwa makolo amene ali ndi kachirombo ka HIV, pali mavuto/nkhawa za padela zomwe amakhala nazo? |
| **Kutseka/ kumaliza** | 1. Zikomo. Awa ndi mafunso amane ndinali nawo kwa inu. Chilipo chili chonse chomwe mukufuna kudziwa zokhuzana ndi ukadawulo/luso lanu mundondomekoyi/thandizoli kapena mmene ogwira ntchito mchipatala angathandizikire kuti apitilize kupereka thandizori kwa ana ongobadwa kumene? |  |
